# Supplementary material for: On the complexity of quantum link prediction in complex networks
Source: Sci Rep. 2024 Jan 10;14:1026. doi: 10.1038/s41598-023-49906-4 (PMC10781705; doi:10.1038/s41598-023-49906-4)
Supplement: Supplementary file 1 — Supplementary Information. [file 41598_2023_49906_MOESM1_ESM.pdf]

Supplementary Material for main article: On the complexity of quantum link prediction in complex networks

João P. Moutinho,[1,2] Duarte Magano,[1,2] and Bruno Coutinho[2]

[1] *Instituto Superior Técnico, Universidade de Lisboa, Portugal*

[2] *Instituto de Telecomunicações, Lisboa, Portugal*

I. CLASSICAL MATRIX POWER ALGORITHMS

A. Deterministic Algorithms

As discussed in the main text, path-based link prediction often requires computing powers of the adjacency matrix in order to find high-valued entries. In this section we discuss classical algorithms for this task, both deterministic and randomized. Let us start with the simple cases of computing A^2 or A^3. If A is sparse enough, it may be advantageous to compute these matrices by explicitly counting all paths of length 2 and 3 in the graph, as written in Algorithms 1 and 2.

For the sake of simplicity, we did not exploit the fact that A is symmetric. Both of these algorithms must access the full graph, and thus they have query complexity $O(Nk_{\mathrm{av}}) = O(|E|)$, corresponding to the number of neighbour queries required to write the graph to memory. In addition to the number of queries, we can also characterize the time complexity given the number of extra operations required, corresponding to the nested loops that run through all paths of the given length in the graph. It was shown in Ref. [1] that

$$\sum_{i,j}^{N} (A^n)_{ij} \leq N\langle k^n \rangle \tag{1}$$

where the equality holds for $n = 2$, and $\langle k^n \rangle$ is the n-th moment of the degree distribution in the graph,

$$\langle k^n \rangle = \frac{1}{N}\sum_{i=1}^{N} k_i^n. \tag{2}$$

From Eq. 1 it follows that the time complexity of Algorithm 1 is $O(N\langle k^2 \rangle)$, and $O(N\langle k^3 \rangle)$ for Algorithm 2.

Algorithm 1: A^2 Counting

Data: Graph $\mathcal{G}(V, E)$
Result: A^2
$(A^2)_{ij} = 0$ **forall** $i, j = 1, ..., N$
forall $v \in \mathcal{V}$ **do**
  forall $i \in \Gamma(v)$ **do**
    forall $j \in \Gamma(v)$ **do**
      $(A^2)_{ij} = (A^2)_{ij} + 1$
    end
  end
end

Algorithm 2: A^3 Counting

Data: Graph $\mathcal{G}(V, E)$
Result: A^3
$(A^3)_{ij} = 0$ **forall** $i, j = 1, ..., N$
forall $v \in \mathcal{V}$ **do**
  forall $u \in \Gamma(v)$ **do**
    forall $i \in \Gamma(v)$ **do**
      forall $j \in \Gamma(u)$ **do**
        $(A^3)_{ij} = (A^3)_{ij} + 1$
      end
    end
  end
end

B. Sampling Algorithms

Instead of deterministically computing A^n to find high-valued entries, however, we may consider algorithms that sample an entry (i, j) of A^n with probability

$$\mathcal{P}[(i,j),\, A,\, n] = \frac{|(A^n)_{ij}|}{\|A^n\|_{1,1}} \tag{3}$$

where $\|.\|_{1,1}$ denotes the $L_{1,1}$ matrix norm. For A^2 and A^3 we write Algorithms 3 and 4, inspired by Refs. [2] and [3], respectively.

Let us start by analysing Algorithm 3 to sample entries

Algorithm 3: A^2 Sampling [2]

Data: Graph $\mathcal{G}(V, E)$
Result: Pair (i, j)

foreach $v \in V$ compute $\tilde{p}_v = k_v^2$;
normalize $p_v = \tilde{p}_v / \sum_v \tilde{p}_v$
select $v \in V$ with probability p_v
randomly select $i \in \Gamma(v)$
randomly select $j \in \Gamma(v)$

of A^2. The first step computes the distribution

$$p_v = k_v^2 / \sum_{v=1}^{N} k_v^2 \tag{4}$$

over all nodes $v \in V$, so that we may then sample a node $v \in V$ from this distribution. The quantity k_v^2 counts the number of paths of length 2 going through node v, disregarding direction and repetition of starting and ending nodes. Then, a pair (i, j) is chosen where both i and j are independent random samples from the neighbourhood of v. Thus, the probability to pick a certain (i, j) conditional on v is

$$\mathcal{P}[(i,j)|v] = \frac{1}{k_v}\frac{1}{k_v} \tag{5}$$

Finally, the probability of sampling a pair (i, j) can be computed by summing over all nodes v in the graph,

$$\mathcal{P}[(i,j)] = \sum_{v=1}^{N} \mathcal{P}[v]\mathcal{P}[(i,j)|v] \tag{6}$$

$$= \sum_{v \in \Gamma(i) \cap \Gamma(j)} \frac{k_v^2}{\sum_{v'}^{N} k_{v'}^2} \frac{1}{k_v^2} \tag{7}$$

$$= \frac{|\Gamma(i) \cap \Gamma(j)|}{\sum_{v'}^{N} k_{v'}^2} \tag{8}$$

$$= \frac{|(A^2)_{ij}|}{\|A^2\|_{1,1}}, \tag{9}$$

where we defined the set of common neighbours between i and j as $\Gamma(i) \cap \Gamma(j)$, and used the following relations: $\mathcal{P}[(i,j)|v] = 0$ for all $v \notin \Gamma(i) \cap \Gamma(j)$, $(A^2)_{ij} = |\Gamma(i) \cap \Gamma(j)|$, and $\sum_v^N k_v^2 = \sum_{i,j}^N (A^2)_{ij} = \|A^2\|_{1,1}$.

Algorithm 4 follows a similar reasoning to sample entries from A^3. The first step computes the distribution

$$p_{uv} = k_u k_v / \sum_{v=1}^{N} \sum_{u \in \Gamma(v)} k_u k_v \tag{10}$$

so that a link (u, v) may be sampled according to this distribution. The quantity $k_u k_v$ counts the number of

Algorithm 4: A^3 Sampling [3]

Data: Graph $\mathcal{G}(V, E)$
Result: Pair (i, j)

foreach $v \in V$
  foreach $u \in \Gamma(v)$ compute $\tilde{p}_{uv} = k_u k_v$;
end
normalize $p_{uv} = \tilde{p}_{uv} / \sum_{uv} \tilde{p}_{uv}$
select (u, v) with probability p_{uv}
randomly select $i \in \Gamma(u)$
randomly select $j \in \Gamma(v)$

paths of length 3 going through link (u, v), starting in a neighbour of u and ending in a neighbour of v. Then, a pair (i, j) is chosen where i is a random sample from the neibhourhood of u and j is a random sample from the neighbourhood of v. The probability to pick a certain (i, j) conditional on (u, v) is

$$\mathcal{P}[(i,j)|(u,v)] = \frac{1}{k_u k_v}. \tag{11}$$

We now define the set

$$L3(i,j) = \{(u,v) : (i,u,v,j) \text{ is a path in } \mathcal{G}\}, \tag{12}$$

which implies that $(A^3)_{ij} = |L3(i,j)|$. Thus, following Algorithm 4, the probability to sample a certain pair (i, j) is

$$\mathcal{P}[(i,j)] = \sum_{(u,v) \in L3(i,j)} \mathcal{P}[(u,v)]\mathcal{P}[(i,j)|(u,v)] \tag{13}$$

$$= \sum_{(u,v) \in L3(i,j)} \frac{1}{k_u k_v} \frac{k_u k_v}{\sum_{v'=1}^{N} \sum_{u' \in \Gamma(v')} k_{u'} k_{v'}} \tag{14}$$

$$= \frac{|(A^3)_{ij}|}{\|A^3\|_{1,1}}. \tag{15}$$

To compute the initial distributions p_v and p_{uv}, both Algorithms 3 and 4 must perform one degree query for each $v \in V$, having query complexity $O(N)$. Algorithm 4 must also access each neighbour of v individually, raising the query complexity to $O(|E|)$. For each query one additional operation is used to compute the entries of p_v and p_{uv}, and thus the time complexity for this step is the same as the query complexity.

Once the distributions p_v and p_{uv} are computed, Algorithms 3 and 4 can produce multiple samples of links (i, j). First, algorithm 3 samples a node v with probability p_v, and Algorithm 4 samples a link (u, v) with probability p_{uv}. This can be described as the general

Complexity		Deterministic	Randomized
Queries	A^2	$O(\|E\|)$	$O(N+n_s)$
	A^3	$O(\|E\|)$	$O(\|E\|)$
Operations	A^2	$O(N\langle k^2\rangle)$	$O(N+n_s\log N)$
	A^3	$O(N\langle k^3\rangle)$	$O(\|E\|+n_s\log\|E\|)$

TABLE I. Complexity comparisons between deterministic and randomized algorithms for A^2 and A^3. Deterministic algorithms output the full matrix, while randomized output n_s samples of links (i,j) with probability proportional to $|(A^n)_{ij}|$.

problem of sampling an entry i of a vector $p\in\mathbb{R}^{N'}$ with probability proportional to $|p_i|$. To solve it, we first build a vector $\hat{p}\in\mathbb{R}^{N'}$, corresponding to the cumulative sum of entries in p,

$$\hat{p}_i=\sum_{j=0}^{i}p_j \tag{16}$$

and then randomly pick a value $0\leq x\leq\hat{p}_{N'}$. Finally, we bisect $\hat{p}$ to find the smallest entry i such that $x<\hat{p}_i$. The entry i corresponds to our sample of p.

Building $\hat{p}$ requires $O(N')$ sums and bisecting $\hat{p}$ has an additive $O(\log N')$ cost. As such, processing p_v and p_{uv} in Algorithms 3 and 4 maintain their respective time complexities of $O(N)$ and $O(|E|)$. Then, producing each sample of v and (u,v) requires $O(\log N)$ and $O(\log|E|)$ operations, respectively. No additional queries to the input are required.

Finally, having sampled v in Algorithm 3 and (u,v) in Algorithm 4, a link (i,j) is sampled by randomly selecting nodes from the neighbourhood of v or (u,v), which we consider to be $O(1)$ operations. We note also that this final step takes no additional queries to the input in Algorithm 4, as the whole graph has already been queried during the processing of p_{uv}. However, for Algorithm 3, each sample of a link (i,j) requires two queries to the neighbourhood of the selected v. Repeated samples may end up reading the whole graph, at which point no new queries are required.

In summary, drawing n_s samples of links (i,j) following Algorithm 3 costs $O(N+n_s)$ queries to the input, up to a maximum of $O(Nk_{\mathrm{av}})=O(|E|)$, and takes $O(N+n_s\log N)$ operations. Drawing n_s samples of links (i,j) following Algorithm 4 costs $O(|E|)$ queries to the input and takes $O(|E|+n_s\log|E|)$ operations. We present in Table I a comparison of both deterministic and randomized algorithms in terms of their query and time complexity.

[1] Miguel Angel Fiol and Ernest Garriga. Number of walks and degree powers in a graph. *Discrete Mathematics*, 309(8):2613–2614, 2009.

[2] Comandur Seshadhri, Ali Pinar, and Tamara G Kolda. Triadic measures on graphs: The power of wedge sampling. In *Proceedings of the 2013 SIAM international conference on data mining*, pages 10–18. SIAM, 2013.

[3] Madhav Jha, C Seshadhri, and Ali Pinar. Path sampling: A fast and provable method for estimating 4-vertex subgraph counts. In *Proceedings of the 24th international conference on world wide web*, pages 495–505, 2015.

[4] Paul Erdős and Alfréd Rényi. On random graphs i. *Publicationes mathematicae*, 6(1):290–297, 1959.

[5] Réka Albert and Albert-László Barabási. Statistical mechanics of complex networks. *Reviews of modern physics*, 74(1):47, 2002.

[6] Jesper Dall and Michael Christensen. Random geometric graphs. *Physical review E*, 66(1):016121, 2002.

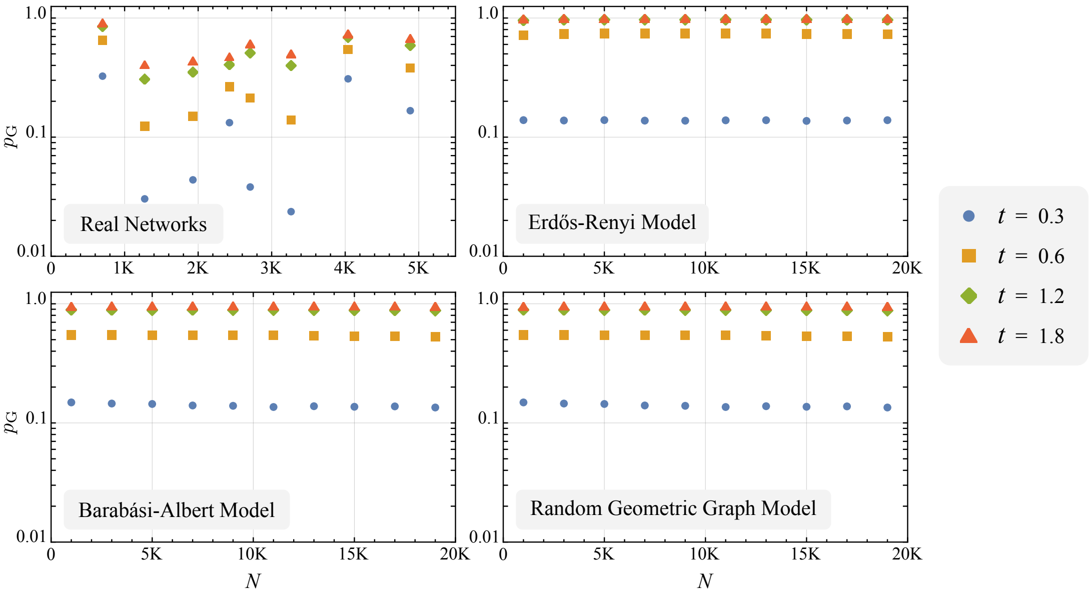

FIG. 1. **Probability of sampling a useful link in QLP vs N.** We compare the variation in the probability of sampling a useful link with QLP with the size of the network N in a range of real and synthetic networks. As N increases, p_G does not show a tendency to decrease in the real networks tested. To compare, we tested three different synthetic network models: the Erdös-Renyi Model [4], the Barabási-Albert Model [5], and the Random Geometric Graph Model [6]. In all cases we fixed $k_{av} = 10$ and simulated QLP for different valuse of t, observing that p_G remains exactly constant as N increases.

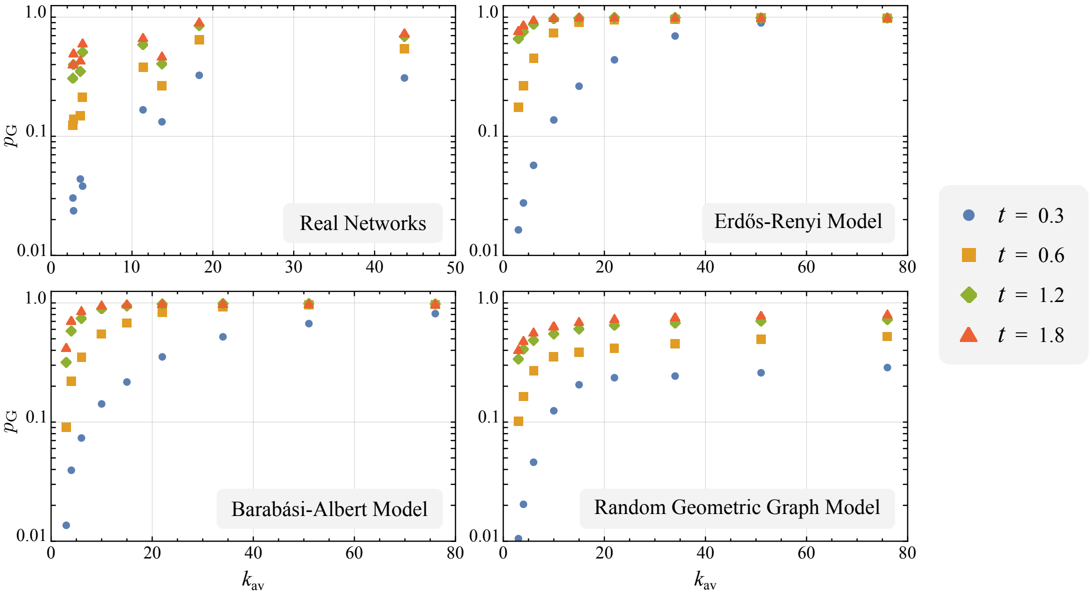

FIG. 2. **Probability of sampling a useful link in QLP vs k_{av}.** Repeating the procedure of Fig. 1, we fixed $N = 5000$ and observed a similar behaviour in the synthethic and real networks: p_G initially grows for increasing k_{av}, and then remains approximately constant.

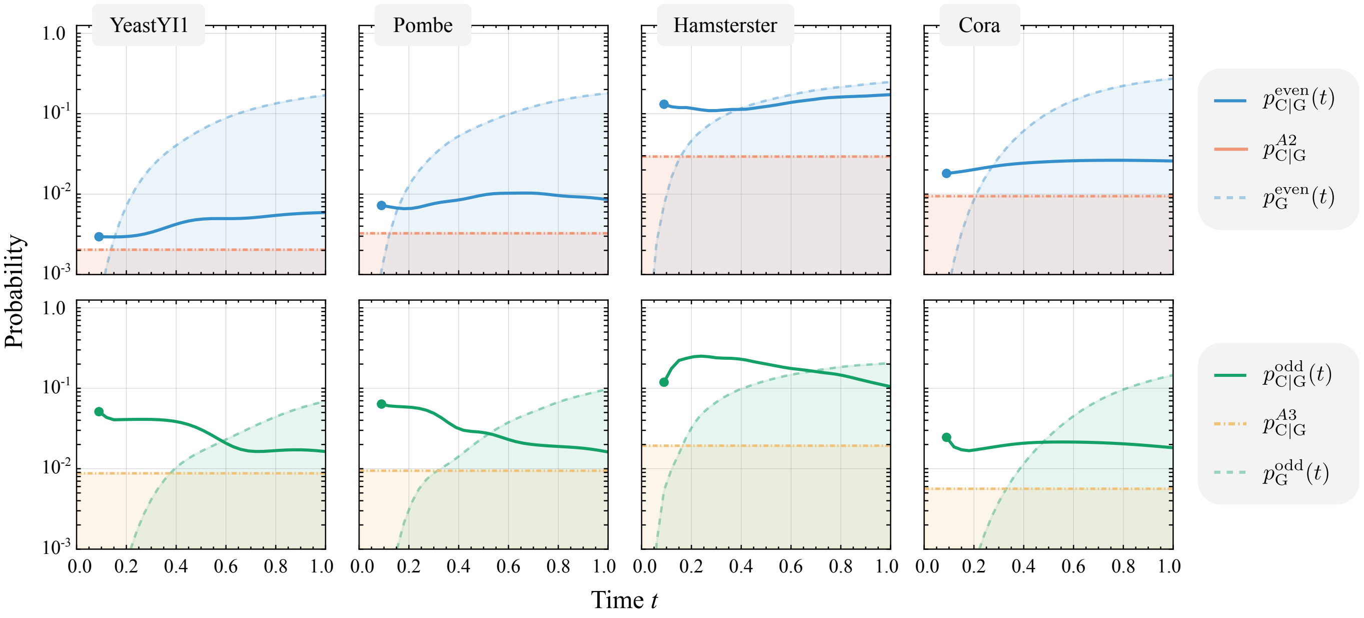

FIG. 3. **Extra results for probability of sampling a correct link in QLP compared to A^2 and A^3.** We compare the precision of QLP with A^2 and A^3 by comparing the probability of sampling a correct prediction given that the sample was useful for four extra networks. The results indicate that a value $t = O(1)$ can be chosen such that $p^{\text{even}}_{C|G}(t) \geq p^{\text{A2}}_{C|G}$ and $p^{\text{odd}}_{C|G}(t) \geq p^{\text{A3}}_{C|G}$ while maintaining a small useful sample overhead given by $1/p_{\text{G}}$. The plots for $p^{\text{even}}_{C|G}(t)$ and $p^{\text{odd}}_{C|G}(t)$ start at $t \approx 0.1$ to avoid the region of small p_{G} where the divisions are numerically unstable, as described in the main text.
